# Methodological Clarifications and Generalizing From Weibo Data. Comment on “Nature and Diffusion of COVID-19–related Oral Health Information on Chinese Social Media: Analysis of Tweets on Weibo”

**DOI:** 10.2196/26255

**Published:** 2021-05-21

**Authors:** Om Prakash Yadav

**Affiliations:** 1 Division of Community Health and Humanities Memorial University of Newfoundland St John's, NL Canada

**Keywords:** COVID-19, dentistry, oral health, dental health, online health, social media, tweet, Weibo, China, health information

I read the article regarding the nature and diffusion of COVID-19–related oral health information conducted by Tao et al [1] with great interest. Information, education, and communication are the best tools to prevent and control any disease. The paper described the temporal and geographic distribution of COVID-19–related oral health information circulated on the social media platform Weibo in China. The study’s focus was to assess COVID-19–related oral health information and explore public reaction.

The introduction was built on existing literature describing COVID-19, its impact on dental practice and specific concerns, and the necessity of information on social media. The authors presented the need for the study in this section; however, it could be elaborated to better understand the target audience.

The authors implemented their research with reproducible methods and described all necessary steps, including screening criteria, piloting and reliability assessment, and statistical methods. The authors included 4 search keywords (“pneumonia of unknown cause,” “coronavirus,” “COVID-19,” and “epidemic”) for COVID-19; however, only 2 keywords (“stomatology” and “dentistry”) were selected to search for oral health and disease-related information. The authors could have included at least 2 additional popular keywords (“oral examination” and “oral health”) to make the search more comprehensive [2,3]. The authors included 15,900 tweets, which strengthened the precision and power of the study.

The study results were well described and presented in a logical and comprehensible manner using tables and graphs. However, some inconsistencies were observed in the x-axis parameters in some figures, and one typo was observed in one of the tables’ data. The authors could also have highlighted the point related to the supply and demand for dental health services during the epidemic.

The authors mentioned some limitations, such as the Weibo platform being more popular among younger age groups than older ones, the nonavailability of information regarding individual users’ characteristics, and the nonavailability of information on online consultation quality services. However, users’ education, academic background, job profile, socioeconomic status, past dental treatment experience, and status of existing oral health problems are essential to categorize and associate oral health information within different parameters.

Other limitations include the use of a single social media source and data collection within a short period of time. These limitations can affect the generalizability of the study. Despite the discussed limitations, the authors are to be congratulated for their novel enrichment of evidence through this exploratory research, and with great excitement, I anticipate future research to highlight further circulation of COVID-19–related oral information carried out in a broader time frame and extended to various geographical locations.

## References

[1] Tao Zhuo-Ying, Chu Guang, McGrath Colman, Hua Fang, Leung Yiu Yan, Yang Wei-Fa, Su Yu-Xiong (2020). Nature and Diffusion of COVID-19–related Oral Health Information on Chinese Social Media: Analysis of Tweets on Weibo. J Med Internet Res.

[2] Park Jung-Eun, Cho Ja-Won, Jang Jong-Hwa (2020). Keyword Trends for Mother-Child Oral Health in Korea Based on Social Media Big Data from Naver. Healthc Inform Res.

[3] LaLonde K (2020). COVID-19 Literature Searches. Medical Library Association.

